# Development of an online public health curriculum for medical students: the public health commute

**DOI:** 10.1186/s12909-019-1734-4

**Published:** 2019-08-03

**Authors:** Sarah Godfrey, Katherine Nickerson, Jonathan Amiel, Benjamin Lebwohl

**Affiliations:** Columbia Vagelos College of Physicians and Surgeons, 630 W 168th St., New York, NY 20032 USA

**Keywords:** Public health, Medical education, Social determinants of health, Minority health, Healthcare disparities

## Abstract

**Background:**

As public health becomes increasingly central to the practice of medicine, educational efforts are necessary to prepare medical students to apply public health concepts in their care of patients. There are few accessible and informative tools to prepare students to engage with population health challenges.

**Methods:**

We distributed an online questionnaire to clinical students, querying gaps in their education on public health topics. Based upon the responses, we developed a web-based curriculum for medical students rotating at a public safety-net hospital on pediatrics, medicine, primary care, psychiatry, and surgery services from April–December 2017 (available at www.publichealthcommute.com). Students received guiding questions and media-based resources (e.g. podcasts, TedTalks, YouTube videos) in weekly modules addressing topics in public health. Each module incorporated 30 min of mobile-optimized content, including specific data relating the topic to the Central Harlem community. Familiarity with public health was assessed with pre- and post-program quizzes, including 10 multiple-choice and 2 open-ended questions.

**Results:**

Among the 70 participating students, 59 (84%) completed both the pre- and post-assessments. The five-week curriculum covered health systems, social determinants, race, substance use, violence, and alternative care models. After completing the five-week curriculum, the mean correct score on a multiple-choice quiz rose from 57 to 66% (*p* = 0.001). In the qualitative section of the test, students were asked what public health topics should be taught in medical school. Frequently suggested topics included social determinants of health (25%), epidemiology (25%), health systems (25%), insurance (21%), policy (17%), economics (17%), racism (15%), and health disparities (8%). When asked how public health will impact their medical career, students frequently responded that it would greatly impact their clinical practice (49%), choice of residency program (17%), and decision to pursue advocacy or additional degrees (15%).

**Conclusions:**

Learners participating in this five-week online public health curriculum demonstrated a significant increase in public health knowledge. The online format allowed for high participation across five different specialty rotations, and community-specific data allowed students to recognize the importance of public health in medical practice.

## Background

As the field of medicine has begun to recognize the effects of psychosocial determinants on health outcomes, formal training in public health has become essential for healthcare providers across disciplines [1, 2]. In order to train effective providers and advocates for underserved patient populations, medical schools are starting to incorporate public health and social medicine into the medical curriculum [3–5].

Some of the reported methods of integrating population health into medical education have included community-based research projects, preclinical didactics and seminar courses, and global health electives [6–8]. However, there remains a dearth of publicly available and easily accessible public health education resources for medical students. This isolates public health discourse to individual institutions and restricts curriculum implementation to already-limited elective or preclinical classroom time.

While multiple medical schools that have instituted in-person public health curricula [6–8], to our knowledge flipped classroom models with community-specific topics have not been reported. The flipped classroom model allows students to interact with digital modules on their own time, which would allow for integration of public health curriculum into the clinical year when psychosocial determinants of health are most relevant to the learner [9]. In this way, students could review a module on a public health theme as they encounter real-life patients affected by that topic. Although a virtual public health curriculum would be useful to students across their medical school tenure, integration into the third year of medical school engenders recognition of the importance of public health topics across multiple medical specialties. In addition, an online-based curriculum provides the opportunity for teaching relevant public health topics catered to students at multiple rotation sites with differing patient populations. Given the support for flipped classroom design in teaching biomedical concepts in medical school, there appears to be an opportunity to expand this model to population health topics [9].

To fill this gap, we designed a novel online media-based public health curriculum for Columbia University medical students rotating at Harlem Hospital during their major clinical year. Our program aimed to provide students with a working knowledge of the health policy and social determinants most relevant to the Central Harlem community, assess student attitudes towards public health, and provide context for caring for an underserved patient population at a local public safety-net hospital.

## Methods

### Needs assessment

Medical students at Columbia Vagelos College of Physicians and Surgeons rotate through local community hospitals, including Harlem Hospital, during their major clinical year for rotations in internal medicine, pediatrics, surgery, primary care, and psychiatry. Students are assigned to rotation sites through a lottery system, and in 2017, 59% (*n* = 95) of all medical students rotated at Harlem Hospital at least once during their major clinical year. In student feedback from those rotations, there was a perceived gap in understanding and preparation for working with an underserved community at a public hospital. To formally assess the students’ needs, we distributed a survey to all clinical students who rotated at Harlem Hospital within the past two years regarding their experiences at Harlem Hospital. Of the approximately 200 students who received the survey, 41 (21%) responded.

Students were asked “what stood out to you relating to the public health concerns of patients at Harlem Hospital?” In their free-text responses, common themes included poverty and inability to afford care (*n* = 14, 34%), poor health literacy (*n* = 12, 29%), lack of resources (*n* = 10, 24%), barriers to health care access (*n* = 8, 20%), housing instability (*n* = 7, 17%), race (n = 7, 17%), environmental exposures (*n* = 6, 15%), substance use (n = 6, 15%), and mental health (*n* = 5, 12%). Echoing many of these responses, one student wrote, “There is an overwhelming fragility of support at Harlem – this is true for the patients who face the daily struggles of being poor, under or uninsured, generally not white…[and] those who work in the hospital.”

Students were then asked about their preparedness to learn about or address their patients’ public health needs. Students frequently responded that they had limited knowledge of resources at a public hospital (*n* = 14, 34%), the social needs of the patient population (*n* = 13, 32%), or health systems (*n* = 5, 12%). Based upon this experience, students were asked what topics would be most useful in a public health curriculum. They expressed the most interest in learning about social determinants (*n* = 28, 68%), health systems (*n* = 25, 61%), substance use (*n* = 22, 54%), race (*n* = 19, 46%), and violence (*n* = 9, 22%). Further, students were asked whether they would and how frequently they were interested in participating in a virtual public health curriculum. The majority of students responded twice weekly (*n* = 12, 29%) or weekly (*n* = 21, 51%) with only 2 students (5%) responding they had no interest.

### Course content

In response to the needs assessment, we created the Public Health Commute (PHC), a 5-week online public health curriculum (available at www.publichealthcommute.com). Recognizing the existing workload for clinical students, we aimed to create an accessible and entirely media-based curriculum (e.g. podcasts, TedTalks, YouTube videos). We created modules that incorporated a total of 30 min of mobile-optimized media content on a particular public health theme that students could conveniently interact with during their free time or the commute to Harlem Hospital. The public health topics also included optional resources (e.g. documentaries, interviews) for students interested in a deeper look at that subject, as well as specific data from the New York City Department of Health relating the topic to the Central Harlem community.

The PHC incorporated the most commonly requested public health themes from the needs assessment: health systems, social determinants of health, race and health, injury and violence, substance use and harm reduction, and alternative models of care. To focus on the material most relevant to each specialty, the topic for the fourth week of the five-week curriculum varied by rotation: (1) injury and violence for surgery and pediatrics students and (2) substance use and harm reduction for medicine, primary care, and psychiatry students. Table 1 lists the module topics and learning objectives for the curriculum. Operating without additional funding, the resources for the modules were compiled from existing publicly available content over the course of a one-month period of investigation, curation, and web design. Specific resources were then reviewed by multiple medical educators, including the faculty advisors and individual clerkship directors, prior to release of the final curriculum.

**Table 1:** Module Topics and Learning Objectives for the Public Health Commute, an Online Public Health Curriculum Offered to Clinical Medical Students at Columbia College of Physicians and Surgeons, New York, New York, 2017

Week	Topic	Learning Objectives
1	Health Systems	• Describe health systems
		• Discuss basics of health economics
		• Define safety-net hospital category
2	Social Determinants of Health	• Define social determinants
		• Discuss trends in healthcare disparities
		• Relate economic inequality to mediators of health outcomes
3	Race and Health	• Demonstrate racial health disparities
		• Define implicit bias
		• Discuss race-based medicine and practice
4	Injury and Violence (pediatrics and surgery rotations)	• Demonstrate rates of gun violence
		• Relate gun violence to public health
		• Discuss self-injury and suicide
4	Substance Use and Harm Reduction (medicine, primary care, and psychiatry rotations)	• Describe the opioid epidemic
		• Define harm reduction
		• Outline the history of U.S. drug policy
5	Alternative Health Systems and Current Health Policy	• Describe healthcare in other countries
		• Explain current U.S. health policy

### Learner characteristics and assessment

Between April 1 and December 22, 2017, 70 medical students rotating at Harlem Hospital were eligible to participate in the PHC curriculum. This included 23 students rotating in internal medicine (33%), 18 in pediatrics (26%), 11 in surgery (16%), 9 in primary care (13%), and 9 in psychiatry (13%). All 70 eligible students were asked to complete pre-and-post curriculum assessments, which included the same ten multiple-choice knowledge-based questions reflecting the course content. The assessments also incorporated two open-ended questions to assess student attitudes towards public health, querying what topics they believed should be taught in medical school and how public health would impact their medical career. Free responses were coded based upon categories of public health topics and areas of influence on their career. As an educational initiative that did not collect human data, this project was exempt from the institutional IRB ethics approval.

### Data analysis

Data for each participant were collected at the beginning and end of the curriculum. Effects of medical specialty and time of year upon change in score were assessed using a repeated measures ANOVA. We used the paired t-test to determine whether there was a statistically significant difference in assessment scores between the pre-and-post curriculum groups. All reported *p* values are 2-sided. The data were analyzed using SPSS 24.0 for Windows (IBM, Armonk, New York).

## Results

Between April and December 2017, all 70 eligible students were invited to participate in the curriculum. Of these 70 students, 59 (84%) completed both the pre-and-post assessments. Participation rates varied by rotation specialty: 100% (*n* = 18) for pediatrics, 89% (*n* = 8) for primary care, 89% (n = 8) for psychiatry, 83% (*n* = 19) for internal medicine, and 55% (*n* = 6) for surgery.

### Knowledge scores

In the pre-curriculum quiz, the mean correct score was 57% of ten multiple choice questions. After completing the five-week curriculum, the mean correct score was 66% (mean difference by paired t-test 8.6, 95%CI 3.6–13.7, *p* = 0.001). Of the 59 students who completed the curriculum, 32 (54%) improved their score by a mean difference of 23.8% (*p* < 0.001). Neither medical specialty nor rotation group was significantly associated with differences between pre-and post-quiz scores (*p* > 0.1).

### Attitude assessment

In the qualitative section of the pre-quiz assessment, students were asked what public health topics should be taught in medical school. Frequently suggested topics included social determinants (*n* = 18, 27%), health systems (*n* = 13, 19%), insurance (*n* = 11, 16%), health disparities (*n* = 9, 13%), policy (*n* = 8, 12%), race (*n* = 6, 9%), and nutrition (n = 6, 9%). After completing the curriculum, the frequency of some suggested topics increased: social determinants (42%, *n* = 25), health systems (30%, n = 18), race (21%, *n* = 12), and substance use (12%, *n* = 7). Table 2 outlines the frequency of suggested public health topics.

**Table 2:** Suggested Public Health Topics for Medical School Curriculum from Pre-and-Post Assessments of Public Health Commute Offered to Medical Students at Columbia College of Physicians and Surgeons, New York, New York, 2017

Topic	Pre-Quiz Number of Responses (out of 67)	Post-Quiz Number of Responses (out of 59)
Social Determinants of Health	18 (27%)	25 (42%)
Health Systems	13 (19%)	18 (30%)
Insurance	11 (16%)	9 (15%)
Health Disparities	9 (13%)	8 (13%)
Health Policy	8 (12%)	3 (4%)
Epidemiology	6 (9%)	5 (9%)
Race	6 (9%)	12 (21%)
Nutrition	6 (9%)	5 (9%)
Preventative Health	5 (7%)	4 (7%)
Women’s Health	4 (6%)	3 (4%)
Violence	4 (6%)	4 (7%)
Substance Use	2 (3%)	7 (12%)
Other	10 (15%)	10 (16%)

When asked how public health will impact their medical career in the pre-quiz, students responded that it would greatly impact their clinical practice (*n* = 22, 33%), patients’ health outcomes (*n* = 14, 21%), dedication to advocacy or working with underserved communities (*n* = 8, 12%), and choice of residency program or employment site (*n* = 7, 10%). After completing the curriculum, a greater proportion of students acknowledged the impact on their clinical practice (*n* = 21, 36%), patient’s outcomes (*n* = 18, 30%), and choice of residency program or employment site (*n* = 10, 16%).

## Discussion

After the first 9 months of curriculum implementation, analysis suggests the Public Health Commute resulted in a statistically significant increase in public health knowledge amongst medical students rotating at a public safety-net hospital. The online media-based format allowed for a high participation rate (84%) across 5 different specialty rotations. After completing the 5-week curriculum, most students (54%) improved their knowledge scores, and many recognized the importance of public health concepts in their clinical practice (36%) and patients’ health (30%). Further, the curriculum incorporated the topics students identified as the greatest gaps in their public health education: health systems, social determinants of health, race and health, injury and violence, substance use and harm reduction, and alternative models of care.

In this study, we formally assessed learning outcomes (Kirkpatrick Level 2) but not reaction, behavior, or results (Kirkpatrick Levels 1, 3 and 4) [10]. We received positive anecdotal feedback from students and faculty members, but future work will include formal program evaluation and quality improvement based upon that information and assessment of how gains in public health knowledge change behaviors and skills in the clinical setting. Nonetheless, our post-intervention test and questionnaire data suggest that an online media-based public health curriculum can influence knowledge and attitudes. As a result, the curriculum has now been expanded to internal medicine, primary care, and pediatrics rotations at all Columbia-affiliated hospitals, including New York Presbyterian Hospital, Harlem Hospital, the Bronx VA, the Allen Hospital, and Stamford Hospital with population-specific data for each site. We anticipate launching additional public health modules designed for obstetrics and gynecology, neurology, and surgery rotations over the next year. To ensure the program’s sustainability, additional medical students have been recruited to create and edit course content, and a faculty advisor supports the project within the medical school curriculum.

As a pilot project, our study has some limitations based on its small sample size. Our initial needs assessment only had a 21% response rate. While respondents represented all of the available class years and rotations, the initially reported gaps in public health knowledge may not represent the entire student population. Further, our initial cohort only included 70 participants with 59 (84%) completing the curriculum at a single medical school, which may limit our ability to generalize the results of this study to other programs. As the curriculum is now a requirement for all clinical year students, future work will include knowledge assessments and data collection for the entire medical student body. Finally, the knowledge assessments were only conducted after finishing the entire curriculum, which limits the evaluation of each individual module.

## Conclusions

In summary, we have designed a novel five-week online media-based public health curriculum for medical students rotating at a public hospital in their major clinical year. The participation rate was high, and students expressed positive reactions to the curriculum. In addition, participating students demonstrated a statistically significant increase in public health knowledge and recognized the importance of public health education in medical school and their future practice of medicine. This virtual public health curriculum is publicly available and easily reproducible for other patient populations, and it could represent a cost effective and engaging approach for integrating population health into medical schools more broadly. As the field of medicine continues to evolve to address social determinants of health, this approach to public health didactics may be a useful tool broadly applicable to undergraduate medical education.

## Data Availability

All data generated or analyzed during the current study are available from the corresponding author on reasonable request.
